# Improved survival of locoregional-advanced larynx and hypopharynx cancer patients treated according to the DeLOS-II protocol

**DOI:** 10.3389/fonc.2024.1394691

**Published:** 2024-06-11

**Authors:** Gunnar Wichmann, Theresa Wald, Markus Pirlich, Matthaeus Stoehr, Veit Zebralla, Thomas Kuhnt, Nils Henrik Nicolay, Peter Hambsch, Irene Krücken, Karl-Titus Hoffmann, Florian Lordick, Regine Kluge, Susanne Wiegand, Andreas Dietz

**Affiliations:** ^1^ Clinic for Otolaryngology, Head and Neck Surgery, University Hospital Leipzig, Leipzig, Germany; ^2^ Clinic for Radiation Oncology, University Hospital Leipzig, Leipzig, Germany; ^3^ Department of Pathology, University Hospital Leipzig, Leipzig, Germany; ^4^ Department of Neuroradiology, University Hospital Leipzig, Leipzig, Germany; ^5^ Department of Medicine, Division of Oncology, University Cancer Center (UCCL), University Hospital Leipzig, Leipzig, Germany; ^6^ Department of Nuclear Medicine, University Hospital Leipzig, Leipzig, Germany

**Keywords:** head neck squamous cell carcinoma (HNSCC), larynx cancer, Hypopharynx cancer, treatment outcome, adjuvant chemotherapy, radiation therapy, radiochemotherapy, larynx organ preservation

## Abstract

**Introduction:**

Larynx organ preservation (LOP) in locoregional-advanced laryngeal and hypopharyngeal squamous cell carcinoma (LA-LHSCC) being only R0-resectable (clear margins > 5 mm) by total laryngectomy (TL) is desirable. Based on tumor-specific survival (TSS) and overall survival (OS) data from the RTOG 91-11 trial and meta-analyses of randomized clinical trials (RCTs), cisplatin-based concurrent radiochemotherapy (CRT) is discussed being superior to cisplatin-based induction chemotherapy followed by radiotherapy (IC+RT) and TL followed by postoperative RT (TL+PORT) or radiochemotherapy (TL+PORCT). Outside of RCTs, T4 LHSCC treated with TL+PORCT demonstrated improved OS and TSS compared to CRT alone; comparisons with docetaxel plus cisplatin (TP)-based IC+RT are unpublished. Head-to-head comparisons in RCTs of these four alternatives are missing.

**Materials and methods:**

We utilized monocentric registry data to compare the outcome in the LOP trial DeLOS-II (NCT00508664) and propensity score (PS)–matched LHSCC patients. DeLOS-II utilized endoscopic tumor staging after one cycle of TP-based IC for selecting TL+R(C)T for non-responders versus IC+RT for responders. Main risk factors for survival (localization hypopharynx, T4, N+, tobacco smoking >30 pack years, alcohol consumption >60 g/day, age, sex) were used to calculate the individual PS for each DeLOS-II patient and 330 LHSCC patients suitable for DeLOS-II according to eligibility criteria in Leipzig by CRT (78), TL+PORT (148), and TL+PORCT (104). We performed PS matching with caliper width 0.2.

**Results:**

The 52 DeLOS-II patients (whole intent-to-treat cohort) and three PS-matched cohorts (52 LHSCC patients each) had equal distribution regarding risk factors including Charlson comorbidity score (CS; all *p* > 0.05) but differed in outcome. During 12,498.6 months of follow-up, 162 deaths (36/41/43/42 in DeLOS-II/TL+PORCT/TL+PORT/CRT, *p* = 0.356) occurred; DeLOS-II patients had superior OS and TSS. Compared to DeLOS-II, the HR (95% CI) observed in TL+PORCT, TL+PORT, and CRT for OS and TSS were 1.49 (0.92–2.43), 1.49 (1.15–3.18), and 1.81 (1.11–2.96) for OS; and 2.07 (0.944–4.58), 3.02 (1.32–6.89), and 3.40 (1.58–7.31) for TSS.

**Conclusion:**

In addition potential LOP, LA-LHSCC suitable for LOP according the DeLOS-II protocol may achieve improved survival.

## Introduction

Larynx organ preservation (LOP) in locoregional-advanced (LA) laryngeal and hypopharyngeal squamous cell carcinoma (LHSCC) that can only be surgically treated by total laryngectomy (TL) is very desirable but is discussed as potentially being more harmful for the patient compared to early TL followed by either adjuvant postoperative radiotherapy (Op+PORT) or platinum-based postoperative radiochemotherapy (Op+PORCT). Two alternative multimodal LOP approaches, either induction chemotherapy (IC) followed by radiotherapy (IC+RT) or platinum-based concurrent radiochemotherapy (CRT), are possible LOP options. Although the German guideline on diagnosis, treatment, and follow-up of laryngeal cancer already recommends IC+RT as treatment option for laryngeal cancer amenable to TL responding to IC (1), IC+RT still remains experimental and is furthermore investigated in clinical LOP trials (2). CRT is established as standard treatment for LOP in LHSCC stages III, IVA, and IVB in many countries including the United States of America and the European Union, for instance The Netherlands. Recently updated results from the most visible randomized clinical trials (RCT), RTOG 91–11, demonstrate improved 10-year OS in the IC+RT arm (38.8% vs. 27.5% in CRT) (3, 4). However, failure of either IC+RT or CRT puts the patient at risk not only regarding the side effects of an ineffective treatment but also the risk associated with prolonged delay of radical salvage treatment and exposure to the further growing tumor, and complications related to late salvage TL (LSTL), for example, wound healing problems or exacerbating comorbidities reducing the chance for salvage surgery per se (5). Another issue is dysphagia and loss of larynx organ function more frequently being associated with CRT.

As failure of CRT requiring LSTL after completed treatment is more often observed in category T4 tumors, and outcome of patients with T4 LHSCC treated by CRT was shown to be inferior compared with counterparts receiving TL+PORCT (6), LOP attempts by CRT should not be recommended for all T4 LHSCC. Inferior outcome in LOP trials and clinical LOP routine is observed in LHSCC with locoregional neck metastasis (N+), T4 tumors, and hypopharyngeal localization of the primary tumor in particular (7). In 2017, we published the outcome of 52 LHSCC patients of the German LOP trial DeLOS-II, the subgroup that was treated in our University Hospital (8). Contrary to the prior findings which led to the recommendations made by the Larynx Preservation Consensus Panel (7), the benefit from treatment according to the DeLOS-II protocol was not limited to smaller LHSCC (T3). This also applies to T4, N+ with more than two involved neck nodes, and in particular hypopharynx cancer whenever the response to the first cycle induction chemotherapy (IC-1) with docetaxel and cisplatin (TP) is sufficient according to the DeLOS-II Early Response Evaluation (≥ 30% endoscopic assessed surface shrinkage of the primary lesion [ETSS ≥ 30%]). However, a newly developed score identified in this investigation may allow for improved patient selection achieving benefit from this LOP approach (8, 9). The LFS score consists of four independent parameters to predict LFS in early responders (endoscopic estimated surface shrinkage = ETSS ≥ 30% estimated 25 ± 4 days after first cycle IC). These are (1) the number of clinically positive nodes (cutoff, 2); (2) the residual primary tumor volume (cutoff, 20%); (3) the residual total tumor volume (cutoff, 5.6 mL); (4) the ratio of residual standard-uptake value (SUV) in [^18^F]-FDG-PET-CT imaging SUV_max_ and SUV_mean_ (cutoff, 1.51). In the formula, each parameter is weighted by its hazard ratio (HR) (12, 6, 5, and 4, respectively); LFS-score ≤ 16 predicts increased LFS, OS, and tumor specific survival (*p* < 0.05) (2, 8, 9). As the overall survival (OS) and tumor-specific survival (TSS) of our DeLOS-II patients (8) was better than outcome in stages III, IVA, and IVB (solely based on N3, not including T4b primaries) LHSCC patients treated in our University Hospital with Op+PORCT and Op+PORT (10), we were interested in the reasons behind those differences and hypothesized that (i) treatment of only by TL curatively resectable LHSCC according to the DeLOS-II protocol could be superior to other treatment modalities, (ii) participation in a randomized clinical trial with eligibility criteria might be responsible for superior outcome in DeLOS-II patients, (iii) time-dependently further improved decision-making processes, superior radiologic diagnostic procedures, and so forth may have substantially contributed to these differences, and (iv) the benefit from DeLOS-II could be different for particular LHSCC subgroups. To clarify these questions and to gain evidence for health decision making (11), addressing patients with risk factors for reduced outcome (hypopharynx cancer, T4, daily alcohol consumption > 60 g/d, tobacco smoking history > 30 pack years), we decided to perform two sets of analyses (1), comparison of the DeLOS-II patients with all locoregional-advanced LHSCC treated in our University Hospital between 1993 and 2016, and (2) comparison of propensity score (PS)–matched LHSCC patients with identical characteristics that are only different regarding the applied treatment.

## Materials and methods

**Figure 1:**
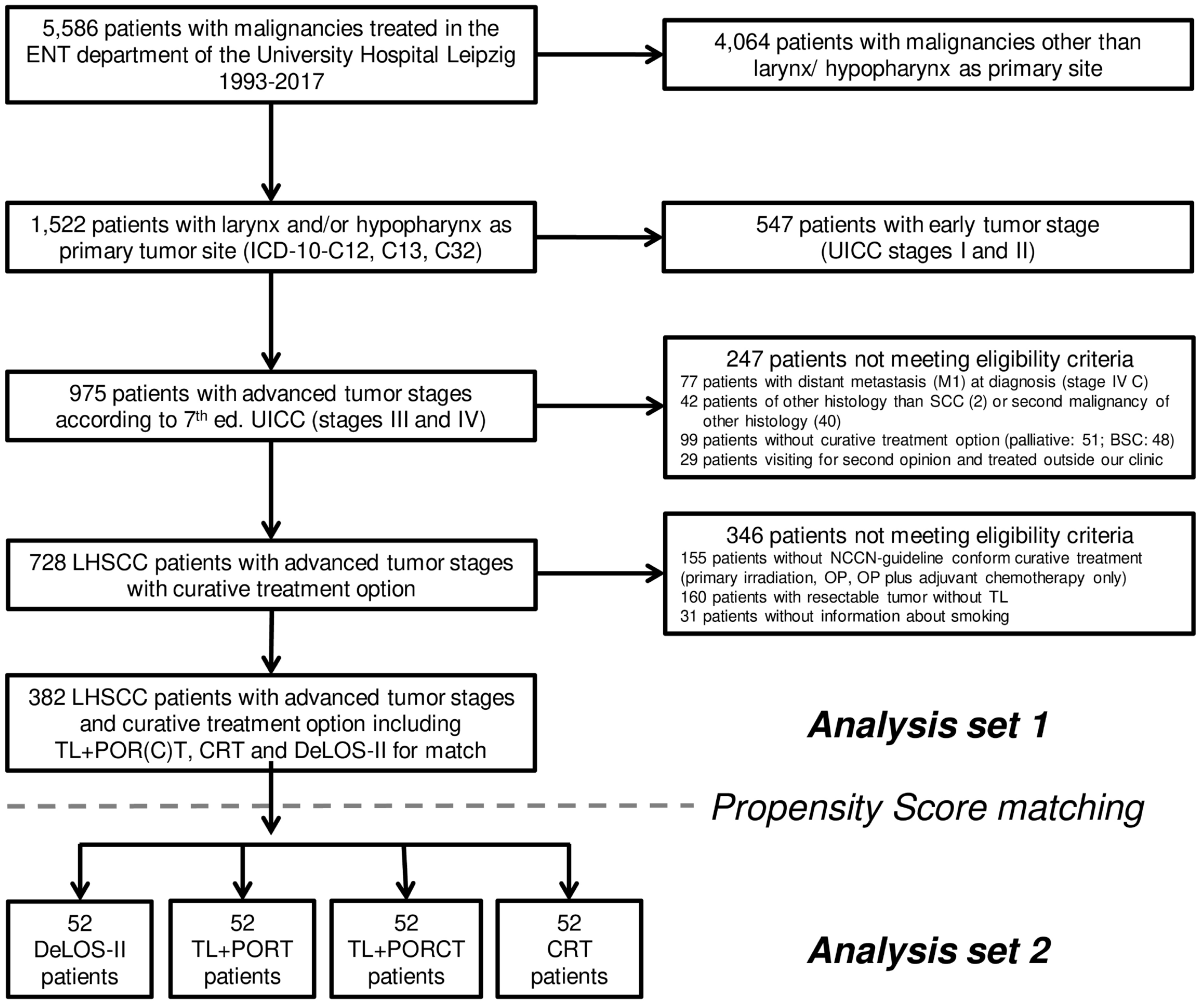
CONSORT diagram showing the selection process from registry data for the two comparisons with the sub-cohort of *n* = 52 DeLOS II-patients. SCC, squamous cell carcinoma; BSC, best supportive care; LHSCC, larynx/hypopharynx SCC; TL, total laryngectomy; PORT, postoperative radiotherapy; PORCT, postoperative radiochemotherapy; CRT, primary concurrent radiochemotherapy.

The clinical data of all cancer patients admitted to the ear, nose, and throat (ENT) department of the University Hospital Leipzig are routinely documented in the tumor data base of the ENT department and additionally submitted to a clinical cancer registry implemented in the Leipzig county area (“Gießen tumor documentary system” [GTDS]). **Figure 1** shows the CONSORT diagram for selection process of LHSCC patients for the comparison. According to their ICD-10 (International Statistical Classification of Diseases and Related Health Problems) codes 1,552 patients with C12, C13, and C32 (carcinoma of the larynx and/or hypopharynx as primary tumor site) were extracted. After exclusion of patients not meeting selection criteria (other histological cancer entities or first presentation with metastatic disease [UICC stage IVC], no curative treatment option due to extension of disease or reduced general health [BSC and palliative therapy], early stage [UICC stage I or II]), a subgroup of 728 patients with locoregional-advanced (LA-) LHSCC (UICC stages III and IV) including the *n* = 52 DeLOS-II patients could be selected for the present analyses.

DeLOS-II was an open-label phase IIB RCT aiming on LOP in only by TL resectable advanced LHSCC by applying a short-induction chemotherapy followed by endoscopic tumor-surface shrinkage (ETSS) estimation 3 weeks after the first cycle (IC-1) of 75 mg/m^2^ docetaxel (T) and cisplatin 75 mg/m^2^ (P) based IC. Patients achieving ETSS ≥ 30% received further two cycles IC, whereas to poor responding LHSCC patients early salvage TL was recommended.

### Propensity score matching for bias-reduced analyses

LA-LHSCC comprises a number of subgroups with varying risk factor profiles and need for radical surgery to achieve oncological safety. Especially LHSCC that can be curatively resected (R0 > 5 mm) without TL by either trans-oral laser microsurgery (TLM) or open partial larynx resection and selective neck dissection instead of radical neck dissection may therefore have better outcome. As DeLOS-II addressed only by TL resectable LHSCC, we excluded all those patients either being (1) resectable without TL, (2) not safely resectable (due to infiltration of essential anatomic structures or impossibility to achieve resection with clear margins, R0 with > 5 mm), (3) without risk factors and thus without need for PORT or even PORCT, and (4) patients who did not receive the recommended RT dose as they might have probably been unfit patients (**Figure 1**). As treating LA-LHSCC patients with surgical resection ± neck dissection (Op) or primary radiotherapy (pRT, monomodal) is not standard of care, we hence excluded such cases from this set of analyses. The *n* = 52 intent-to-treat DeLOS-II patients treated in Leipzig were compared with 52 PS-matched patients each of the other three treatment modalities. PS matching allows for reducing inequalities in the distribution of already known or assumed risk factors of highest impact on outcome and can successfully be used for matching between groups (12). Therefore, PS matching is considered a sufficient alternative to RCTs (12). Matching variables here were the localization of the primary lesion (larynx vs. hypopharynx), T4 versus lower T category, N0 versus N+ and N3 versus other N, sex, age (continuous), tobacco smoking (four categories, never vs. < 10 PY vs. 10–29 PY vs. ≥ 30 PY), and daily alcohol consumption (four categories, 0 vs. < 30 g/d vs. 30–60 g/d vs. > 60 g/d). After applying logistic regression for comparison of DeLOS-II patients and all other patients, the weight of each risk factor regarding the possibility of being treated according to the DeLOS-II protocol is assessed. Each patient’s characteristics regarding the matching variables is expressed in a single score, the propensity score (PS). According to recommendations by Kuss et al. (12), an optimal matching algorithm with a caliper width of 0.2 standard deviations of the linear predictor was used to perform a 1:1 PS matching utilizing SPSS version 29.0 (IBM Deutschland GmbH, Ehningen, Germany). By checking the option to prioritize exact matches, a patient with the identical or nearly the same PS was matched to a DeLOS-II patient, and hence subgroups equal in number of patients having an identical risk factor profile could be compared.

### Survival prediction of patients

To confirm the identical characteristics of the PS-matched groups, we calculated predicted survival probabilities for each PS-matched patient according to a nomogram being developed for predicting 2-, 3-, and 5-year survival in patients receiving definitive CRT for locally advanced head and neck squamous cell carcinoma from a secondary analysis of NRG/RTOG 0129, 0522, and 1016 (13, 14). Nomogram criteria were age, sex, race, smoking status, primary site, T, and N categories. Three factors were set to default values as they are unknown to our cohort: p16 status (unknown), anemia (no), and Zubrod performance status (normal activity). We recorded expected OS- and PFS-probabilities as total points as well as predicted 2-, 3-, and 5-year survival probabilities to compare the expected survival in each therapy group and check for possible outcome differences in matching.

### Statistics

Differences between the patient groups were investigated by *Pearson’s* Chi-squared (χ^2^) tests for contingency tables and logistic regression; summarized data from two-sided statistics are shown also after applying correction for *p*-values according to *Bonferroni*. OS was defined as time from diagnosis to death from any cause, censoring patients alive or lost to follow-up at the time of last available information. TSS was defined as time from diagnosis to head neck squamous cell carcinoma (HNSCC)–specific death censoring patients alive or lost to follow-up at the time of last available information or having passed without cancer. Death from other cause or non-cancer–related survival (NCRD) was defined as time from diagnosis to death not caused by any malignancy censoring patients alive or with death caused by HNSCC. Survival analyses were performed by the Kaplan–Meier method applying the log-rank test. A *p* < 0.05 was considered as significant. Cox proportional hazard regression models applying the stepwise forward method were used for multivariate analysis of covariates with *p* < 0.2 in univariate analysis to identify independent predictors (*Pi*) for OS, TSS, and NCRD and their impact on outcome according the HR accompanied by their 95% confidence intervals. Stability of the model was assessed through internal validation by applying the bootstrap with 1 000 iterations as recommended. PS matching and data analysis were performed in SPSS Version 29. For sensitivity analysis regarding residual confounding of outcome through the potential impact of matching covariate, we used heteroscedastic *t*-tests and *Cohen’s d* to compare OS and TSS probabilities of patients in treatment groups according to each patient’s individual survival probability calculated in multivariate Cox proportional hazard regression considering only the matching variables.

## Results

Characterization of the total patient cohort with LA-LHSCC and curative treatment option (*n* = 728) is shown in **Table 1**. Ninety-one percent were male. Mean age was 59.6 years (*SD* = 9.9; range 32.6–87.6). Forty-seven of 728 (6%) never smoked and 117 of 728 (16.1%) did not report prior alcohol consumption. Fifty-seven percent (414 of 728) of the LHSCC were primarily located in the larynx, 43% (314 of 728) in the hypopharynx.

**Table 1:** The characteristics of the total cohort of locoregional-advanced LHSCC patients treated in DeLOS-II or with radiotherapy (RT) only, concurrent radiochemotherapy (CRT), total laryngectomy followed by postoperative radiotherapy (TL+PORT) or by postoperative radiochemotherapy (TL+PORCT), and other therapy protocols (e.g., surgery only) are shown (total *n* = 728).

Characteristics			Total	
			(*N* = 728)	
Age (years)				
< 50		*n* (%)	125	(17%)
50–59		*n* (%)	249	(34%)
60–69		*n* (%)	219	(30%)
≥ 70		*n* (%)	135	(19%)
Sex				
Male		*n* (%)	659	(91%)
Female		*n* (%)	69	(9%)
Tobacco smoking				
0 pack years		*n* (%)	47	(6%)
≤ 30 pack years		*n* (%)	114	(16%)
> 30 pack years		*n* (%)	567	(78%)
never		*n* (%)	47	(6%)
former		*n* (%)	109	(15%)
current		*n* (%)	550	(76%)
missing		*n* (%)	22	(3%)
Alcohol consumption				
0 g/d		*n* (%)	117	(16%)
> 0–30 g/d		*n* (%)	170	(23%)
30–60 g/d		*n* (%)	131	(18%)
> 60 g/d		*n* (%)	265	(36%)
missing		*n* (%)	45	(6%)
Tumor location, stage				
Hypopharynx		*n* (%)	314	(43%)
Larynx		*n* (%)	414	(57%)
T1		*n* (%)	27	(4%)
T2		*n* (%)	118	(16%)
T3		*n* (%)	263	(36%)
T4a		*n* (%)	296	(41%)
T4b		*n* (%)	24	(3%)
N0		*n* (%)	219	(30%)
N1		*n* (%)	120	(16%)
N2a		*n* (%)	20	(3%)
N2b		*n* (%)	188	(26%)
N2c		*n* (%)	135	(19%)
N3		*n* (%)	46	(6%)
Stage II (UICC)		*n* (%)	1	(0%)
Stage III (UICC)		*n* (%)	191	(26%)
Stage IVA (UICC)		*n* (%)	470	(65%)
Stage IVB (UICC)		*n* (%)	66	(9%)
Therapy				
DELOS-II		*n* (%)	52	(7%)
TL+PORT		*n* (%)	253	(35%)
TL+PORCT		*n* (%)	184	(25%)
CRT		*n* (%)	82	(11%)
RT		*n* (%)	73	(10%)
other		*n* (%)	84	(12%)

The mean follow-up time of the total cohort was 92.1 (95% CI: 82.1–102.1) months, median follow-up time was 40.4 (95% CI: 34.6–46.1) months. The longest follow-up period was 344.2 months, while its minimum (due to the intent-to-treat inclusion of DeLOS-II patients and a therapy-related death shortly after first cycle IC) was 0.4 months. Mean OS was 87.15 (95% CI: 77.9–96.4) months, and median OS was 40.1 (95% CI: 34.96–45.2) months. Mean TSS was 130.8 (95% CI: 116.4–145.2) months and median TSS was 69.7 (95% CI: 53.5–85.9) months.

### Outcome in unmatched patients

After exclusion of 346 patients without according to NCCN-guideline recommended curative treatment (i.e., either primary irradiation, OP or OP plus adjuvant chemotherapy missing simultaneous radiation; *n* = 155), a possibility of tumor-resection without TL (*n* = 160) or patients without information about smoking history (*n* = 31), 330 patients remained for PS matching to the 52 patients treated according to the DeLOS-II protocol.

As the outcome of DeLOS-II patients was significantly improved compared to these 330 LA LHSCC patients with respect to OS (*p* = 0.0213) and TSS (*p* = 0.0003) without observed differences related to other causes of death (*p* = 0.4830), we performed a sensitivity analysis to avoid over-interpretation of findings potentially related to a number of sources of bias. Among the well-known risk factors and covariates with high impact on OS and TSS are age, sex, tobacco smoking, alcohol consumption, and the localization of the primary tumor as well as T category, N category, and UICC stage. The characteristics of the 330 patients eligible for matching with DeLOS-II patients are shown in **Table 2** within their treatment group (DeLOS-II with *n* = 52, TL+PORT with *n* = 148, TL+PORCT with *n* = 104, and CRT with *n* = 78 patients).

**Table 2:** Characteristics of the patients with locoregional-advanced LHSCC treated in DeLOS-II (*n* = 52) or by total laryngectomy (TL) followed by postoperative radiotherapy (TL+PORT, *n* = 148) or postoperative radiochemotherapy (TL+PORCT, *n* = 104), and primary concurrent radiochemotherapy (CRT, *n* = 78) eligible for propensity score matching are shown (total *n* = 382).

Characteristics		Total		DeLOS-II		TL+PORT		TL+PORCT		CRT		*P*-value
		(*N* = 382)		(*n* = 52)		(*n* = 148)		(*n* = 104)		(*n* = 78)		
		*n*	(%)	*N*	(%)	*n*	(%)	*n*	(%)	*n*	(%)	
Age (years)												
< 50		78	(20%)	14	(27%)	21	(14%)	27	(26%)	16	(21%)	**0.034**
50–59		155	(41%)	23	(44%)	55	(37%)	44	(42%)	33	(42%)	
60–69		112	(29%)	10	(19%)	50	(34%)	30	(29%)	22	(28%)	
≥ 70		37	(10%)	5	(10%)	22	(15%)	3	(3%)	7	(9%)	
Sex												
male		343	(90%)	45	(87%)	135	(91%)	93	(89%)	70	(90%)	0.626
female		39	(10%)	7	(13%)	13	(9%)	11	(11%)	8	(10%)	
Tobacco smoking												
0 pack years		17	(4%)	0	(0%)	6	(4%)	6	(6%)	5	(6%)	0.455
≤ 30 pack years		175	(46%)	25	(48%)	62	(42%)	52	(50%)	36	(46%)	
> 30 pack years		190	(50%)	27	(52%)	80	(54%)	46	(44%)	37	(47%)	
never		17	(4%)	0	(0%)	6	(4%)	6	(6%)	5	(6%)	0.281
former		48	(13%)	10	(19%)	17	(11%)	15	(14%)	6	(8%)	
current		317	(83%)	42	(81%)	125	(84%)	83	(80%)	67	(86%)	
Alcohol consumption												
0 g/d		62	(16%)	2	(4%)	25	(17%)	20	(19%)	15	(19%)	0.249
> 0–30 g/d		83	(22%)	18	(35%)	30	(20%)	22	(21%)	13	(17%)	
30–60 g/d		90	(24%)	11	(21%)	36	(24%)	24	(23%)	19	(24%)	
> 60 g/d		147	(38%)	21	(40%)	57	(39%)	38	(37%)	31	(40%)	
Tumor location, stage												
hypopharynx		161	(42%)	29	(56%)	37	(25%)	49	(47%)	46	(59%)	**< 0.001**
larynx		221	(58%)	23	(44%)	111	(75%)	55	(53%)	32	(41%)	
T1		2	(1%)	0	(0%)	0	(0%)	1	(1%)	1	(1%)	**< 0.001**
T2		35	(9%)	6	(12%)	7	(5%)	14	(13%)	8	(10%)	
T3		150	(39%)	26	(50%)	68	(46%)	35	(34%)	21	(27%)	
T4a		187	(49%)	20	(38%)	72	(49%)	54	(52%)	41	(53%)	
T4b		8	(2%)	0	(0%)	1	(1%)	0	(0%)	7	(9%)	
N0		119	(31%)	10	(19%)	73	(49%)	19	(18%)	17	(22%)	**< 0.001**
N1		55	(14%)	4	(8%)	25	(17%)	16	(15%)	10	(13%)	
N2a		7	(2%)	0	(0%)	0	(0%)	2	(2%)	5	(6%)	
N2b		95	(25%)	19	(37%)	23	(16%)	30	(29%)	23	(29%)	
N2c		86	(23%)	18	(35%)	23	(16%)	29	(28%)	16	(21%)	
N3		20	(5%)	1	(2%)	4	(3%)	8	(8%)	7	(9%)	
UICC Stage II		1	(0%)	1	(2%)	0	(0%)	0	(0%)	0	(0%)	**< 0.001**
Stage III		89	(23%)	11	(21%)	53	(36%)	14	(13%)	11	(14%)	
Stage IVA		265	(69%)	39	(75%)	90	(61%)	82	(79%)	54	(69%)	
Stage IVB		27	(7%)	1	(2%)	5	(3%)	8	(8%)	13	(17%)	

Bold P-values indicate statistical significance.

The differences between LA LHSCC patients in the treatment groups may reflect prioritization of either patient or treating physicians for one or the other treatment regimen and decision making of the multidisciplinary tumor board but independent from the underlying cause may have had a significant influence on outcome disparities. Especially LHSCC patients receiving CRT had disproportionally higher percentages of hypopharyngeal carcinomas, N+, T4, and especially cT4b primaries (primary tumors judged to be irresectable respective to the R0 > 5 mm criterion for low-risk resection).

As these and other inequalities may have confounded or even caused the significant differences between DeLOS-II patients and the other treatment groups, a data-driven matching approach utilizing SPSS for automatic 1:1 matching of each DeLOS-II patient and one patient each from the other treatment groups according to propensity scores was applied.

### Validating PS matching

Patients characteristics after PS matching (*n* = 208) are shown in **Table 3**. With the distribution in N categories being the only exception, they are equally distributed (all *p* > 0.062). This also relates to the matching criteria mentioned above (*p* > 0.071).

**Table 3:** Characteristics of the four propensity score-matched groups (*n* = 52 each) of locoregional-advanced LHSCC patients treated in DeLOS-II or by total laryngectomy (TL) followed by postoperative radiotherapy (TL+PORT), TL followed by postoperative radiochemotherapy (TL+PORCT), and primary concurrent radiochemotherapy (CRT) are shown (total *n* = 208).

Characteristics	Total		DeLOS-II		TL+PORT		TL+PORCT		CRT		*P-*value	
	(*n* = 208)		(*n* = 52)		(*n* = 52)		(*n* = 52)		(*n* = 52)			
	*n*	(%)	*n*	(%)	*n*	(%)	*n*	(%)	*n*	(%)		
Age (years)												
< 50	51	(25%)	14	(27%)	9	(17%)	18	(35%)	10	(19%)	0.116	
50–59	86	(41%)	23	(44%)	19	(37%)	18	(35%)	26	(50%)		
60–69	46	(22%)	10	(19%)	12	(23%)	12	(23%)	12	(23%)		
≥ 70	25	(12%)	5	(10%)	12	(23%)	4	(8%)	4	(8%)		
Sex												
male	186	(89%)	45	(87%)	48	(92%)	47	(90%)	46	(88%)	0.797	
female	22	(11%)	7	(13%)	4	(8%)	5	(10%)	6	(12%)		
Tobacco smoking												
0 pack years	4	(2%)	0	(0%)	1	(2%)	1	(2%)	2	(4%)	0.891	
≤ 30 pack years	103	(50%)	25	(48%)	26	(50%)	27	(52%)	25	(48%)		
> 30 pack years	101	(49%)	27	(52%)	25	(48%)	24	(46%)	25	(48%)		
never	4	(2%)	0	(0%)	1	(2%)	1	(2%)	2	(4%)	0.651	
former	29	(14%)	10	(19%)	8	(15%)	6	(12%)	5	(10%)		
current	175	(84%)	42	(81%)	43	(83%)	45	(87%)	45	(87%)		
Alcohol consumption												
0 g/d	16	(8%)	2	(4%)	3	(6%)	5	(10%)	6	(12%)	0.621	
> 0–30 g/d	50	(24%)	18	(35%)	13	(25%)	10	(19%)	9	(17%)		
30–60 g/d	51	(25%)	11	(21%)	12	(23%)	14	(27%)	14	(27%)		
> 60 g/d	91	(44%)	21	(40%)	24	(46%)	23	(44%)	23	(44%)		
Charlson score												
0	111	(53%)	34	(65%)	26	(50%)	24	(46%)	27	(52%)	0.223	
>0	97	(47%)	18	(35%)	26	(50%)	28	(54%)	25	(48%)		
Tumor location, stage												
Hypopharynx	111	(53%)	29	(56%)	20	(38%)	33	(63%)	29	(56%)	0.071	
Larynx	97	(47%)	23	(44%)	32	(62%)	19	(37%)	23	(44%)		
T1	2	(1%)	0	(0%)	0	(0%)	1	(2%)	1	(2%)	0.111	
T2	28	(13%)	6	(12%)	3	(6%)	12	(23%)	7	(13%)		
T3	90	(43%)	26	(50%)	28	(54%)	20	(38%)	16	(31%)		
T4	88	(42%)	20	(38%)	21	(40%)	19	(37%)	28	(54%)		
N0	44	(21%)	10	(19%)	19	(37%)	5	(10%)	10	(19%)	**0.009**	
N1	27	(13%)	4	(8%)	10	(19%)	7	(13%)	6	(12%)		
N2a	7	(3%)	0	(0%)	0	(0%)	2	(4%)	5	(10%)		
N2b	61	(29%)	19	(37%)	11	(21%)	16	(31%)	15	(29%)		
N2c	58	(28%)	18	(35%)	11	(21%)	17	(33%)	12	(23%)		
N3	11	(5%)	1	(2%)	1	(2%)	5	(10%)	4	(8%)		
UICC Stage II	1	(0%)	1	(2%)	0	(0%)	0	(0%)	0	(0%)	0.062	
Stage III	45	(22%)	11	(21%)	18	(35%)	7	(13%)	9	(17%)		
Stage IVA	149	(72%)	39	(75%)	33	(63%)	40	(77%)	37	(71%)		
Stage IVB	13	(6%)	1	(2%)	1	(2%)	5	(10%)	6	(12%)		

Bold P-values indicate statistical significance.

### Validation of equal distribution of risk factors in the PS-matched cohort

For external validation of the matching results, we applied the nomogram for predicting 2-, 3-, and 5-year OS and PFS developed by (13) as explained in the Methods section. For each patient, total points for OS and PFS depending on the individual risk factors were calculated. There were no significant differences in mean values in comparisons between patients treated in DeLOS-II or any other therapy protocol applied using heteroscedastic Student’s *t*-test (all *p* > 0.49), and only insignificantly small differences below the lower limit for small effect sizes (i.e., *d _LL_* = 0.2) were identified using Cohen’s *d* (all *d* < 0.11). In univariate analysis of variance, there were also only insignificant differences in total points as well as in 2-, 3-, and 5-year OS and PFS among the four therapy groups. However, by considering only the mean of the predicted OS, the expected order was DeLOS-II > TL+PORT > TL+PORCT > CRT, whereas the expected order in PFS was TL+PORT > DeLOS-II > TL+PORCT > CRT for 2-, 3-, and 5-year survival according to the nomogram. The matching criteria themselves failed to gain significance in multivariate Cox proportional hazard regression models demonstrating absence of a substantial bias from residual confounding. To conclude, an equal distribution of risk factors after PS matching is given in our cohort allowing for further outcome analyses.

### Outcome in PS-matched patients

Within 12,498.6 months of follow-up, 162 deaths occurred in the PS-matched cohort of 208 patients compared. The non-uniform distribution of deaths among PS-matched LHSCC patients that had same characteristics regarding known risk factors used for PS matching also demonstrated improved outcome achieved by applying the DeLOS-II protocol.

**Figure 2:**
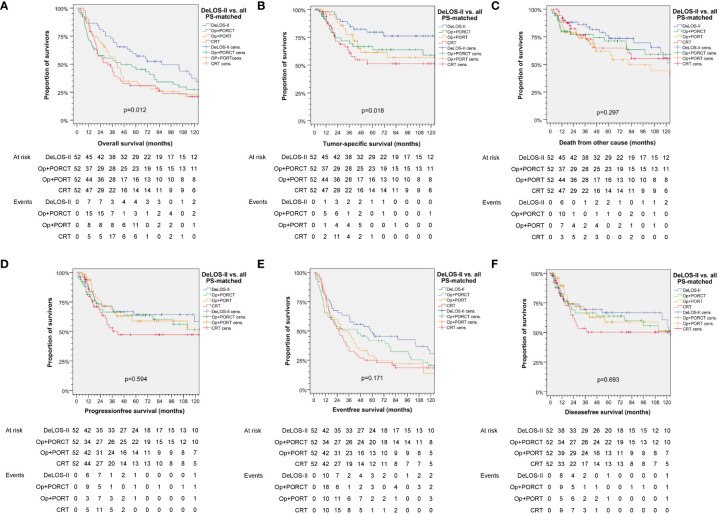
Kaplan–Meier curves for **(A)** overall, **(B)** tumor-specific, **(C)** non-cancer–related, **(D)** progression-free, **(E)** event-free and **(F)** disease-free survival demonstrating superior survival of patients treated according to the DeLOS-II protocol (*n* = 52) compared to PS-matched patients treated with total laryngectomy (TL) followed by postoperative radiotherapy (TL+PORT), TL followed by postoperative radiochemotherapy (TL+PORCT), or primary concurrent radiochemotherapy (CRT) (*n* = 52 each). *P*-values shown are from log-rank tests. Op = TL.

Censoring for 125 months, OS and TSS in LHSCC patients treated by TL+PORT, TL+PORCT and CRT differed significantly from those treated according to the DeLOS-II protocol (*p* = 0.012 for OS and *p* = 0.018 for TSS). Comparing all four therapy regimens, DeLOS-II patients had superior OS and TSS (as shown in **Figure 2**), but this was not always significantly better and with decreasing effect over time, compared to LHSCC patients treated by TL+PORCT (*p* = 0.133 and *p* = 0.070), TL+PORT (*p* = 0.018 and *p* = 0.091), and CRT (*p* = 0.011 and *p* = 0.006).

Detailed survival data censored 125 months after date of primary diagnosis including the absolute survival rate and relative gain in percentage for OS and TSS for DeLOS-II at various time points are shown in **Table 4**.

**Table 4:** Detailed survival data [overall (OS) and tumor-specific survival (TSS) with mean, corresponding 95% confidence interval (CI), median] censored at 125 months as well as absolute survival rates (%) and relative gain (%) achieved by using the DeLOS-II cohort at 12, 36, 60, and 120 months are shown for the four propensity score-matched groups (*n* = 52 each) of the locally advanced LHSCC patients treated in DeLOS-II or by total laryngectomy (TL) followed by postoperative radiotherapy (TL+PORT), TL followed by postoperative radiochemotherapy (TL+PORCT), and primary concurrent radiochemotherapy (CRT).

	Mean (months)	95% CI (months)	*P-*value	Median (months)	12 months		36 months		60 months		120 months	
					ASR	relative Gain	ASR	relative Gain	ASR	relative Gain	ASR	relative Gain
Overall survival												
DeLOS-II	77.8	64.9–90.6	Ref.	86.2	86.5%	Ref.	73.1%	Ref.	55.8%	Ref.	23.1%	Ref.
TL+PORCT	60.0	46.5–73.4	0.133	44.7	71.2%	21.6%	53.8%	35.7%	44.2%	26.1%	21.2%	9.1%
TL+PORT	55.0	42.9–67.1	**0.018**	41.1	84.6%	2.3%	53.8%	35.7%	30.8%	81.3%	15.4%	50.0%
CRT	51.3	39.0–63.6	**0.011**	31.4	90.4%	-4.3%	42.3%	72.7%	26.9%	107.1%	11.5%	100.0%
Tumor-specific survival												
DeLOS-II	103.9	92.3–115.5	Ref.	NR	86.5%	Ref.	73.1%	Ref.	55.8%	Ref.	23.1%	Ref.
TL+PORCT	87.0	71.8–102.3	0.070	NR	71.2%	21.6%	53.8%	35.7%	44.2%	26.1%	21.2%	9.1%
TL+PORT	86.9	71.9–102.0	0.091	NR	84.6%	2.3%	53.8%	35.7%	30.8%	81.3%	15.4%	50.0%
CRT	76.2	60.5–92.0	**0.006**	NR	90.4%	-4.3%	42.3%	72.7%	26.9%	107.1%	11.5%	100.0%

P-values shown are from log-rank-tests, bold P-values indicate significant differences compared to treatment according to DeLOS-II protocol. CI, confidence interval; ASR, absolute survival rate; Ref, reference; NR, not reached.

Patients with laryngeal SCC had superior OS and TSS compared to hypopharyngeal SCC patients when treated by OP+PORCT, OP+PORT, or CRT (for OS all *p* < 0.047 and for TSS all *p* < 0.068) but when treated according to the DeLOS-II protocol, there was no difference (*p* = 0.963 and *p* = 0.387). Women had better OS and TSS than men (*p* = 0.018 and 0.002) regardless of therapy regimen. Patients with a cumulative dose of > 30 PY and a daily alcohol consumption > 60 g had worse OS and TSS (all *p* < 0.002). In those patients, the best outcome, however, was achieved when treated according to the DeLOS-II protocol.

Regardless of treatment choice, patients with stage T4 or N2/N3 had worse OS and TSS compared to lower T or N categories (all *p* < 0.001). OS and TSS in stage N2/N3 patients were better when treated by DeLOS-II protocol (*p* = 0.007, *p* = 0.011). Consequently, OS and TSS according to UICC stage was superior in III > IVA > IVB (*p* < 0.001). For UICC stage III, mean OS was 74.0 (95% CI 67.2–80.8) months and median 80.1 (95% CI 65.6–94.6) months; for UICC stage IVA, mean OS was 56.1 (95% CI 51.7–60.5) months and median OS was 36.2 (95% CI 30.9–41.5) months; and for UICC stage IVB, mean OS was 33.8 (95% CI 23.8–43.7) months and median OS 14.1 (95% CI 9.8–18.4) months.

**Figure 3:**
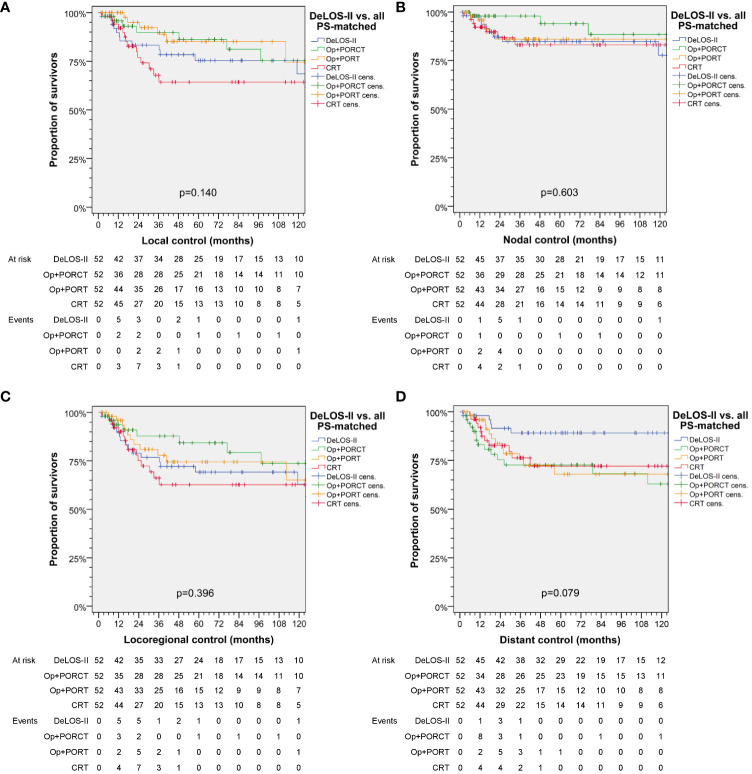
Kaplan–Meier curves showing **(A)** local, **(B)** nodal, **(C)** locoregional and **(D)** distant control of DeLOS-II patients and PS-matched patients with locoregional-advanced LHSCC treated with total laryngectomy (TL) followed by postoperative radiotherapy (TL+PORT), TL followed by postoperative radiochemotherapy (TL+PORCT), or primary concurrent radiochemotherapy (CRT) indicate superior distant control of DeLOS-II patients. *P*-values shown are from log-rank tests. Op = TL.

Patients treated with a surgical approach [TL+POR(C)T] tend to have better local control, whereas nodal control and thus, locoregional control, was slightly superior when treated by TL+PORCT. Local and locoregional control (local and locoregional relapse-free survival) and distant control (distant metastasis-free survival) were found to be comparable among treatments. However, distant control tended to be superior after applying induction chemotherapy according to DeLOS-II (*p* = 0.079). Respective Kaplan–Meier curves are shown in **Figure 3**.

**Figure 4:**
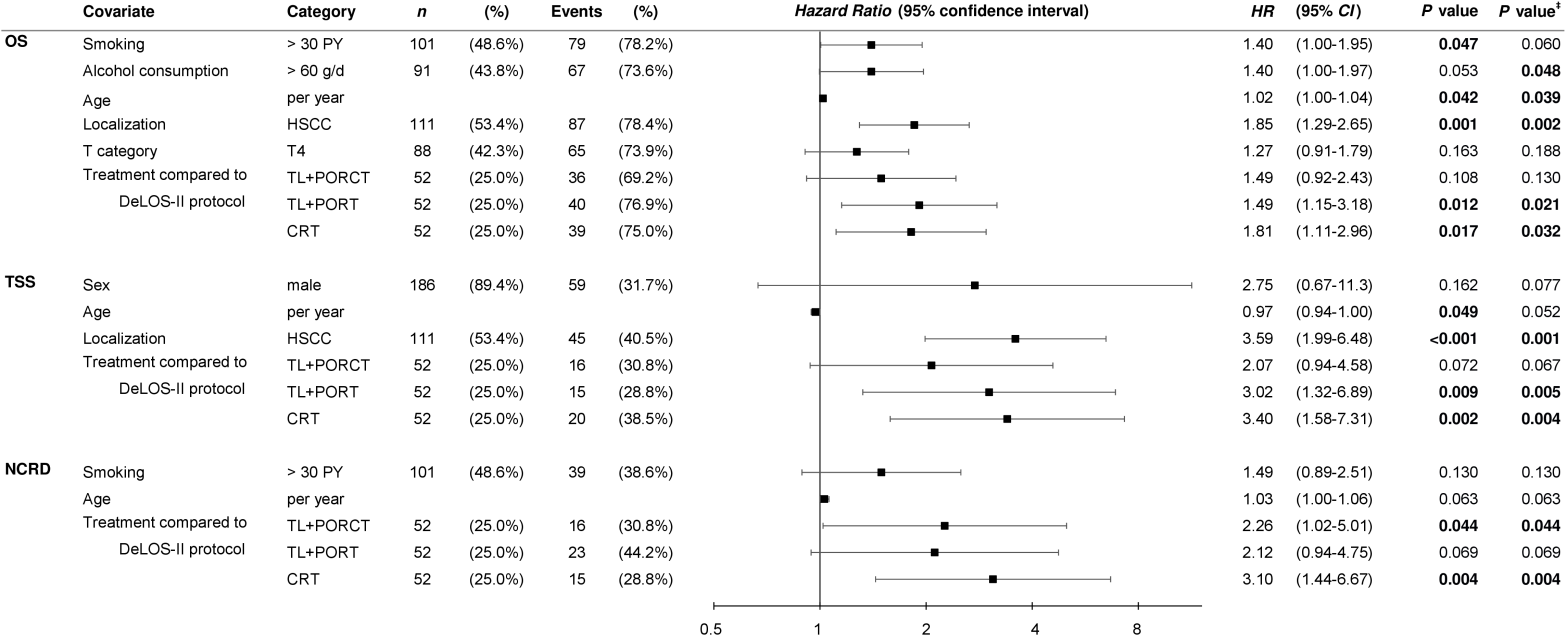
Multivariate Cox proportional hazard regression models built via the step-wise forward likelihood ratio-driven selection of independent predictors of overall (OS), tumor-specific (TSS), and survival time to non-cancer–related death (NCRD) with censored survival time for 125 months are shown. *P*-values shown are two-sided. *P*-value ^‡^ is calculated from bootstrapping using 1 000 iterations. HSCC, hypopharyngeal squamous cell carcinoma; OP, total laryngectomy; PORT, postoperative radiotherapy; PORCT, postoperative radiochemotherapy; CRT, primary concurrent radiochemotherapy; HR, hazard ratio; CI, confidence interval.

For multivariate analyses, Cox proportional hazard regression models were used to identify independent predictors for OS, TSS, and NCRD as shown in **Figure 4**.

Interestingly, despite the absence of residual confounding from disproportional distribution of risk factors used for PS matching, we demonstrated an impact of smoking (> 30 PY) and alcohol consumption (> 60 g/day) on OS, but not on TSS, in the multivariate models, which included the treatment as covariate. This points to an interaction between the lifestyle-associated risk factors and the treatment. However, the localization of the primary in the hypopharynx emerged as the major risk factor for TSS and OS, whereas the competing risk for NCRD was not pertained. While the T4 category had a minor impact on OS but not TSS or NCRD, N categories independent from building particular categories for comparisons were not included in the data-driven built multivariate models. The most relevant finding was, however, to see the treatment regimens applied to emerge as significant *Pi* for survival. Treatment according to DeLOS-II despite inclusion of the full intention-to-treat cohort demonstrated superiority to any other treatment (**Figures 2** **–** **5**; **Supplementary Figure S1**; **Table 4**). Independent from inclusion of the treatment-related early deaths (and potential immortality-time bias potentially present in the three other treatment groups containing only per-protocol treated patients), treatment according to the DeLOS-II protocol was not inferior to TL+PORCT and improved survival compared to TL+PORT and CRT.

**Figure 5:**
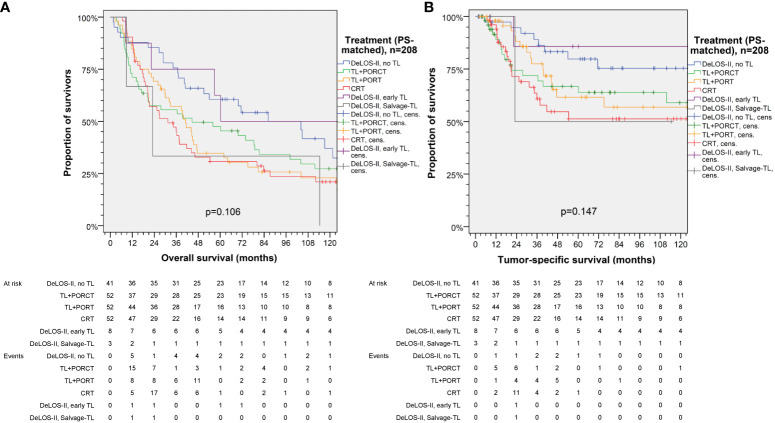
Kaplan–Meier curves for **(A)** overall and **(B)** tumor-specific survival of DeLOS-II patients separately displaying patients receiving early total laryngectomy (TL) and Salvage-TL compared to PS-matched patients with locoregional-advanced LHSCC treated with TL followed by postoperative radiotherapy (TL+PORT), TL followed by postoperative radiochemotherapy (TL+PORCT), or primary concurrent radiochemotherapy (CRT). *P*-values shown are from log-rank tests.

## Discussion

Within this retrospective single-centered cohort study, long-term follow-up data up to 125 months after primary diagnosis of advanced LHSCC (UICC stages III and IV) with a curative treatment option, that is, surgically treatable by total laryngo(pharyng)ectomy, of patients was assessed. To compare survival data of *n* = 52 patients treated in the LOP trial DeLOS-II at the University Hospital Leipzig to survival data of patients treated by TL+PORCT, TL+PORT, and CRT as conventional therapy regimens, PS matching according to main risk factors (localization of the primary lesion, T4 vs. lower T category, N0 vs. N+ and N3 vs. other N, sex, age, tobacco smoking (never vs. < 10 PY vs. 10–29 PY vs. ≥ 30 PY), and daily alcohol consumption (0 vs. < 30 g/d vs. 30–60 g/d vs. > 60 g/d) was performed and validated using *t*-tests and nomograms predicting 2-, 3-, and 5-year OS and PFS.

We demonstrated superior outcome in patients of the DeLOS-II cohort compared to patients with LA-LHSCC treated with TL+POR(C)T or CRT (**Figure 2**). As also patients experiencing early treatment-related death or refusing TL as recommended for non-responders are included in the *n* = 52 DeLOS-II cohort compared to *n* = 52 LHSCC patients with completed therapy options according to NCCN guidelines, this improvement is not limited to the sub-sample of DeLOS-II patients receiving full per-protocol-treatment. The superior OS and TSS censored at 125 months was validated in pairwise comparison of all *n* = 52 DeLOS-II patients with PS-matched LHSCC treated with TL+PORT (*p* = 0.018, *p* = 0.091) and CRT (*p* = 0.011, *p* = 0.006), but not with TL+PORCT (*p* = 0.133, *p* = 0.070) (**Figure 2**; **Table 4**). The positive effect regarding excess in survival of DeLOS-II patients declined over time, as competing causes of death (non-cancer related death) became more influential in patients with advanced age. Given the PS matching procedure and the equal distribution of risk factors for survival, disease-related factors (localization of the primary lesion, T category and N category) as well as patient-related factors (age at diagnosis, sex, alcohol consumption, and PY smoked), the LOP concept of IC followed by RT for responders and early TL for non-responders cannot be judged as a risky approach to circumvent an inevitably required TL. The improved outcome in these 52 DeLOS-II patients shows similar findings as the RTOG 91–11 10-year outcome data (3) and furthermore indicate non-inferiority of the DeLOS-II protocol with IC to CRT as discussed by Licitra et al. (4, 9) as alternative interpretation approach. Overall, the sequential treatment of LHSCC with TP-based IC followed by RT can be recommended independently from probably achievable further improvement in patient selection applying the LFS-score (8), and the demonstration that T4 category, N category and hypopharynx are no limitation regarding a potential benefit from the LOP approach of DeLOS-II (8). This is in sharp contrast to the findings of Rosenthal et al. (6) who showed that T4 larynx cancer patients had reduced outcome if treated with CRT compared to those receiving TL+PORT or TL+PORCT. The comparison of our PS-matched patients receiving TL+PORT or TL+PORCT did not confirm their findings, potentially because of the early response evaluation after IC-1 leading to early instead of delayed salvage TL in non-responders, and the benefit from IC regarding a significantly improved distant metastasis free survival (**Figure 3**), the major reason behind long-term TSS.

Within the past two decades, concepts of treatment of LA-LHSCC shifted towards more standardized use of postoperative (adjuvant) and primary multimodal treatment. Adjuvant radiotherapy (PORT) has been established as standard treatment since many years and was recommended in oncologic risk situations according to pathological examination. Among these are positive resection margins (R1 or R2), perineural invasion (Pn1), lymphovascular invasion (L1), extracapsular spread (ECS), >N1, largest node > 3 cm, and especially if multiple of these risk factors are present (15, 16). The results of the landmark phase-III RCTs performed by the European Organization for Research and Treatment of Cancer (EORTC) (17), Radiation Therapy Oncology Group (RTOG 9501) (18, 19), and radiotherapy cooperative clinical trials group of the German Cancer Society (ARO) (20) unequivocally demonstrating a benefit from platinum-based post-operative radiochemotherapy (PORCT) jointly led to changes in treatment recommendations since 2004 (21). Therefore, the indication to perform PORT alone after resection of LA-LHSCC has switched to simultaneous platinum-based PORCT in patients at “intermediate risk” (R0 < 5 mm) and “high risk” (R1, ECS) for relapse. The surgical concepts for advanced resectable tumors of the oral cavity, oro-/hypopharynx and larynx have not changed significantly within the past 20 years. In experienced centers, additional techniques like trans-oral laser or trans-oral robotic microsurgery (TLM, TORS) to conventional surgical approaches and neck dissection [classified from Robbins (22)] are standard procedures for treatment of advanced resectable HNSCC. These surgical procedures are supplemented by PORT or PORCT depending on risk factors (20). Still, the updated recommendations for irradiation recommend to apply doses exceeding 57 Gy (in ≥ 1.8 Gy daily fractions) to the primary tumor site and a further increase in case of ECS, R1, R2 or L1 (16). Intensity modulated radiotherapy now comprehensively available allows for protection of vulnerable organs (e.g., the salivary glands) and structures and hence further increased intensities. The German S3 guideline (23), for instance, recommends even higher doses of 64–66 Gy for the primary tumor site as well as 54 Gy applied to potentially affected neck regions in the adjuvant setting. The indication for additional adjuvant chemotherapy was comprehensively discussed in several retrospective analyses where factors like “more than three involved lymph nodes”, “pN2b or pN2c category”, “venous infiltration (V1)”, Pn1 and L1 were suggested for indicating PORCT (24–26). Cooper et al. (19) published after analysis of 10-year outcome data of the RTOG 9501 study that only ECS and R1-resection remained as significant risk discriminators. Allover, benefit for OS of additional chemotherapy in all three trials was 7%–13% (EORTC 22931 *HR* 0.70; *p* = 0.04 (17); RTOG 9501 2-year OS *HR* 0.84, *p* = 0.19; 10-year OS 27.1% vs. 19.6%, *p* = 0.07 (18); ARO 96–3 5-year OS 58% vs. 49% (20). Recently, in our earlier proof-of-concept analysis in Germany, the consequent use of standards in PORCT indication showed to significantly increase survival in this indication group comparing outcome data after introduction of PORCT standards after 2004 (10). Moreover, the high-cumulative dose of 300 mg cisplatin (q3w 100 mg/m^2^) as recommended by Cooper and Bernier (18, 24) has raised many problems due to high acute toxicity and need of dose reductions in many patients even within RCT. Therefore, in many centers in the United States and Germany and in the adjuvant setting, the cumulative cisplatin dose was reduced to 200 mg/m^2^, and regimens like weekly cisplatin 40 mg/m^2^ for five cycles (27) or two cycles of five infusions of 20 mg/m^2^ days 1–5 and 29–33 [without 5-FU, modified from Fietkau (20)] became popular in daily routine and many leading centers switched to reduced cisplatin regimens without compromising local and locoregional control.

The subgroup analyses of DeLOS-II patients versus PS-matched patients receiving the other treatment modalities demonstrated the highest gain in TSS and OS in patients with hypopharynx carcinoma, T4 primaries and the highest level of PY smoked (>30 PY) and daily alcohol consumption >60 g/d. Therefore, these high-risk LHSCC patients should not be excluded from LOP trials applying IC+RT. Even more consequently, these patients should receive at least one cycle TP-based IC to evaluate their response and to decide if their LHSCC can be curatively treated by further two IC cycles followed by RT or should be excised by early TL. The non-responders treated by early TL followed by PORT had (compared with responders only just passing the ETSS ≥30%) an improved TSS and OS despite that they had prior to IC-1 larger volumes of the primary and of neck nodes, and especially an insufficient response to IC-1. This means that the non-responders received the individually best treatment and being non-responder does not necessarily mean being at increased risk—provided, the decision for early TL is realized without delay. As all DeLOS-II non-responders had the best TSS it is even more suggested that at least one cycle TP-based IC can improve survival of locoregional-advanced LHSCC that can only be curatively excised by TL. A subgroup analysis comparing early laryngectomized DeLOS-II patients demonstrated survival within the 95% CI of DeLOS-II patients with LOP and above LHSCC undergoing TL+PORCT (**Figure 5**).

For finding the same number of patients within the subgroups of varying treatments, PS matching was used. The use of this matching method maybe or may not be known or intuitively accepted by the readers as an alternative to performing a RCT. However, scientific knowledge cannot be gained only from RCTs; RCTs also have their drawbacks and mostly lack external validity hampering transferability of findings into the routine setting (11). Even more relevant is the need of resources and high cost of RCTs and nearly impossibility to perform RCTs in a particular peculiarity of rare diseases, for example, LHSCC only curatively resectable by TL. The German DeLOS-II trial involved a total of 35 German hospitals and required more than 6 years for accrual of 173 patients and required more than 10 years for publication of the main findings (2, 28). For a quicker implementation of treatment regimens, all available information should be assessed to accelerate the development of an improved therapy regimen for a rare disease, for example, LHSCC. As DeLOS-II is the so far only LOP trial utilizing short-time IC and evaluation of response after IC-1 and achieved LOP in a substantial proportion of patients but also superior outcome regarding TSS and OS, the questions posed in the introduction require answers within reasonably short time. At this time, to the best of our knowledge, the best way to assess the impact of the diagnostic procedures and the treatment regimen utilized by DeLOS-II on TSS and OS of LHSCC was the comparison with matched LHSCC patients. The use of PS matching is an established method for gaining reliable information by comparing only subjects with the same characteristics regarding known confounders and risk factors for the outcome of interest, mostly the treatment modality to which they could have been assigned (12).

The T and N categories of included patients were based on the 7th ed. of the UICC/AJCC staging systems but are congruent with the earlier UICC 6th and 7th ed. used in the 3 NRG/RTOG trials 0129, 0522, and 1016 that were used in the 863MO nomogram. They have high agreement in definitions for T categories but compared to the UICC 8th ed. did not include ECS to define higher N categories and upstaging of patients (29, 30). Therefore, an exact correlation of results cannot be ascertained to the current AJCC 8th ed. but might be considered for LHSCC patients in our study. However, differences in alcohol consumption, tobacco smoking, and age distribution in our LA LHSCC patient cohort and LA HNSCC patients in NRG/RTOG trials 0129, 0522, and 1016 may exist but appear to be negligible with respect to the purpose we used the nomograms. Here, the nomograms demonstrate that there were only slight (insignificant) differences between the PS-matched patients in the four treatment groups in the predicted OS and PFS probabilities no matter if using total points or the predicted 2-, 3-, and 5-year survival probabilities (all *d* < 0.11, *p* > 0.49).

Some weaknesses of our study have to be discussed: the data resulted from a retrospective (unscheduled) analysis of registry data. The study had to face some severe lacks in the documentation of valuable information before 2007 especially regarding relevant prognostic risk factors like ECS, width of resection margins and clear indication for choosing PORT or PORCT as multidisciplinary tumor meetings were not established at that point. Moreover, the heterogeneity of treatment protocols and the different cytostatics used and variable regimens which have been used before 2004 diminishes the comparability of about a quarter each of the cohorts and all together with the sub-cohort of DeLOS-II patients. However, three quarters of patients in the three treatment regimens might have had benefit from advances in head and neck oncology after finalizing DeLOS-II recruitment in 2012. In addition to cisplatin or cisplatin/5FU regimens also carboplatin, paclitaxel and in some patients mitomycin C have been applied in 63.5% of patients before 2004, while the use of these chemotherapeutic drugs decreased to 7.4% after 2004. In other words: in the absence of clear documentation of reasons to choose cisplatin in only one third of patients deemed to require cisplatin-based adjuvant PORCT, potential bias could lead to difficulties in interpretation of findings. Furthermore, documentation of confounding factors like tobacco and alcohol use was taken from the routine record documentation and may be of sometimes limited internal validity.

Nevertheless, the strengths of our study are augmented by a complete follow-up data set based on the intent to treat in a long time period in one academic cancer center. Confounding due to differences in tumor stages, sex and age discrimination as well as alcohol consumption and smoking behavior could be predominantly excluded. The mono-centric nature of our analysis reduced the influence of additional suspected relevant confounders as surgical procedures, hospital environmental factors, and supportive care were highly homogeneous within the observation time. Tumor classification was consistent and clearly defined over the two periods under investigation.

The superior outcome in DeLOS-II patients was validated by analyses of PS-matched patients and verified our findings in the total number of LHSCC patients. Subgroup analyses consistently demonstrated that improved outcome was predominantly based on improved outcome of those patients with highest risk for treatment failure, namely, hypopharyngeal carcinoma, T4 category, and high exposure to alcohol and tobacco smoke. Especially responders, evaluated after IC-1 by endoscopically determined ETSS ≥ 30%, reached excellent outcome. Non-responders who received early TL and adjuvant therapy according to NCCN-guideline standards showed no inferiority in survival compared to conventional treatment regimens. Altogether, induction chemotherapy + radiotherapy following DeLOS-II protocol for LOP was not inferior regarding outcome compared to conventional treatment regimens in a PS-matched cohort of patients with advanced LHSCC.

## Data availability statement

The raw data supporting the conclusions of this article will be made available by the authors, without undue reservation.

## Ethics statement

The studies involving humans were approved by Ethics Committee of the Medical Faculty of the University Leipzig (votes 166-07-12072006, 201–10-12072010, and 202-10-12072010). The studies were conducted in accordance with the local legislation and institutional requirements. The participants provided their written informed consent to participate in this study.

## Author contributions

GW: Conceptualization, Data curation, Formal analysis, Funding acquisition, Investigation, Methodology, Project administration, Resources, Software, Supervision, Validation, Visualization, Writing – original draft, Writing – review & editing. TW: Conceptualization, Data curation, Formal analysis, Investigation, Methodology, Project administration, Resources, Validation, Visualization, Writing – original draft, Writing – review & editing, Software. MP: Writing – review & editing. MS: Writing – review & editing. VZ: Writing – review & editing. TK: Writing – review & editing. NN: Writing – review & editing. PH: Writing – review & editing. IK: Writing – review & editing. K-TH: Writing – review & editing. FL: Writing – review & editing. RK: Writing – review & editing. SW: Writing – original draft, Writing – review & editing. AD: Writing – review & editing, Writing – original draft.

## Supplementary material

Supplementary Figure 1 Kaplan–Meier curves for **(A)** overall survival, **(B)** tumor-specific, and **(C)** non-cancer–related death in 330 locoregional-advanced LHSCC UICC stage III to IVB patients treated according to NCCN guidelines either by total laryngectomy (TL) followed by post-operative radiotherapy (TL + PORT) or radiochemotherapy (TL + PORCT) or larynx-organ preservation approach through concurrent cisplatin-based chemo-radiotherapy (CRT) compared to the whole intent-to-treat cohort of 52 DeLOS-II patients demonstrate significant superior survival of LA LHSCC patients treated according to the DeLOS-II protocol. *P*-values shown are from log-rank tests.
