# Supplementary figures and images for: Improved survival of locoregional-advanced larynx and hypopharynx cancer patients treated according to the DeLOS-II protocol
Source: Front Oncol. 2024 Jun 11;14:1394691. doi: 10.3389/fonc.2024.1394691 (PMC11198870; doi:10.3389/fonc.2024.1394691)

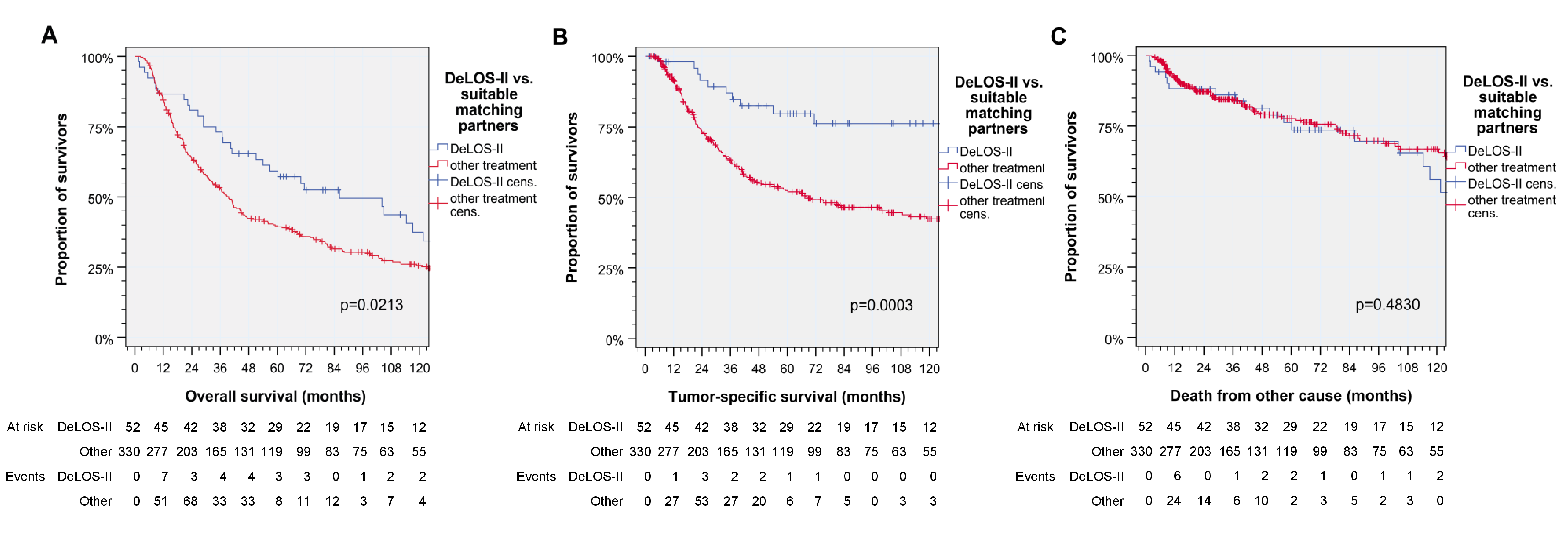

Supplement: Supplementary Figure 1 — Kaplan–Meier curves for (A) overall survival, (B) tumor-specific, and (C) non-cancer–related death in 330 locoregional-advanced LHSCC UICC stage III to IVB patients treated according to NCCN guidelines either by total laryngectomy (TL) followed by post-operative radiotherapy (TL + PORT) or radiochemotherapy (TL + PORCT) or larynx-organ preservation approach through concurrent cisplatin-based chemo-radiotherapy (CRT) compared to the whole intent-to-treat cohort of 52 DeLOS-II patients demonstrate significant superior survival of LA LHSCC patients treated according to the DeLOS-II protocol. P-values shown are from log-rank tests. [file Image_1.tiff]
